# Supplementary material for: The Fml1-MHF complex suppresses inter-fork strand annealing in fission yeast
Source: eLife. 2019 Dec 19;8:e49784. doi: 10.7554/eLife.49784 (PMC6952179; doi:10.7554/eLife.49784)
Supplement: Supplementary file 1. [file elife-49784-supp1.docx]

**Supplementary File 1:** Direct repeat recombinant frequencies

| Genotype and strain number | Number of colonies analysed | Ade^+^ His^-^  (deletion)  recombinant  frequency (x 10^-4^)^a^ | | Ade^+^ His^+^  (gene conversion)  recombinant  frequency (x 10^-4^)^a^ | |
| --- | --- | --- | --- | --- | --- |
|  |  | Mean | *P* value^b^ | Mean | *P* value^b^ |
| wild-type  MCW8020 | 23 | 419.7  (+/- 127.9) | - | 182.8  (+/- 78.6) | - |
| *fml1*∆  MCW8300 | 20 | 1087  (+/- 288.8) | <0.0001^c^ | 64.8  (+/- 26.5) | <0.0001^c^ |
| *fml1*^D196N^  MCW9268 | 18 | 1286  (+/- 339.4) | <0.0001^c^ | 95.1  (+/- 50.0) | <0.001^c^ |
| *fml1*^K99R^  MCW9281 | 16 | 1539  (+/- 605.9) | <0.0001^c^ | 76.3  (+/- 36.4) | <0.0001^c^ |
| *fml1*^∆C^  MCW9266 | 19 | 763.3  (+/- 256.3) | <0.0001^c^ | 64.2  (+/- 41.9) | <0.0001^c^ |
| *rad51*∆  MCW8296 | 22 | 198.0  (+/- 72.16) | <0.0001^c^ | 8.03  (+/- 6.81) | <0.0001^c^ |
| *fml1*∆ *rad51*∆  MCW9496 | 22 | 1659  (+/- 785.0) | <0.0001^c^  <0.0001^d^ | 11.82  (+/- 10.67) | <0.0001^c^  <0.3037^d^ |
| *mhf2*^D87A^  MCW9220 | 17 | 588.7  (+/- 194.4) | <0.01^c^ | 122.6  (+/- 67.5) | <0.05^c^ |
| *fml1*^AAA^  MCW9283 | 16 | 677.6  (+/- 310.8) | <0.001^c^ | 154.6  (+/- 45.7) | 0.2050^c^ |
| *mhf2*^D87A^ *fml1*^AAA^  MCW9269 | 18 | 893.5  (+/- 193.7) | <0.0001^c^ | 99.4  (+/- 40.9) | <0.001^c^ |

^a^ The values in parentheses are the standard deviations about the mean.

^b^ *P* values are derived from the Mann-Whitney U test comparing the mean values as indicated.

^c^ Compared to the equivalent mean recombinant frequency in MCW8020.

^d^ Compared to the equivalent mean recombinant frequency in MCW8296.
