# Supplementary material for: The Fml1-MHF complex suppresses inter-fork strand annealing in fission yeast
Source: eLife. 2019 Dec 19;8:e49784. doi: 10.7554/eLife.49784 (PMC6952179; doi:10.7554/eLife.49784)
Supplement: Supplementary file 2. [file elife-49784-supp2.docx]

**Supplementary File 2:** List of *S. pombe* strains used in this study (in order of appearance)

| Strain | Mating type | Genotype | Source |
| --- | --- | --- | --- |
| MCW8020 | *h^+^* | *ade6-M375 int*::*pUC8/2000 bp spacer/his3^+^/RTS1-AO/ade6-L469 ura4-D18 his3-D1 leu1-32 arg3-D4* | (Morrow et al., 2017) |
| MCW8300 | *h^+^* | *fml1*∆::*natMX4 ade6-M375 int*::*pUC8/2000 bp spacer/his3^+^/RTS1-AO/ade6-L469 ura4-D18 his3-D1 leu1-32 arg3-D4* | (Morrow et al., 2017) |
| MCW9268 | *h^+^* | *fml1*∆::*fml1*^D196N^-*natMX4 ade6-M375 int*::*pUC8/2000 bp spacer/his3^+^/RTS1-AO/ade6-L469 ura4-D18 his3-D1 leu1-32 arg3-D4* | This study |
| MCW9281 | *h^+^* | *fml1*∆::*fml1*^K99R^-*natMX4 ade6-M375 int*::*pUC8/2000 bp spacer/his3^+^/RTS1-AO/ade6-L469 ura4-D18 his3-D1 leu1-32 arg3-D4* | This study |
| MCW9266 | *h^+^* | *fml1*∆::*fml1*^∆C1-603^- *natMX4 ade6-M375 int*::*pUC8/2000 bp spacer/his3^+^/RTS1-AO/ade6-L469 ura4-D18 his3-D1 leu1-32 arg3-D4* | This study |
| MCW8296 | *h^?^* | *rad51*∆::*arg3^+^ ade6-M375 int*::*pUC8/2000 bp spacer/his3^+^/RTS1-AO/ade6-L469 ura4-D18 his3-D1 leu1-32 arg3-D4* | This study |
| MCW9496 | *h^-^* | *fml1*∆::*natMX4 rad51*∆::*arg3^+^ ade6-M375 int*::*pUC8/2000 bp spacer/his3^+^/RTS1-AO/ade6-L469 ura4-D18 his3-D1 leu1-32 arg3-D4* | This study |
| MCW1221 | *h^+^* | *ura4-D18 his3-D1 leu1-32 arg3-D4* | Lab strain |
| MCW2080 | *h^+^* | *fml1*∆::*natMX4 ura4-D18 his3-D1 leu1-32 arg3-D4* | (Sun et al., 2008) |
| MCW6923 | *h^+^* | *fml1*∆::*fml1*^∆C1-603^- *natMX4 ura4-D18 his3-D1 leu1-32 arg3-D4* | This study |
| MCW6976 | *h^+^* | *fml1*∆::*fml1*^AAA^-*natMX4 ura4-D18 his3-D1 leu1-32 arg3-D4* | This study |
| MCW5677 | *h^+^* | *mhf2*∆::*mhf2^+^*-*kanMX6 ura4-D18 his3-D1 leu1-32 arg3-D4* | This study |
| MCW5112 | *h^+^* | *mhf2*∆::*natMX4 ura4-D18 his3-D1 leu1-32 arg3-D4* | This study |
| MCW6376 | *h^+^* | *mhf2*∆::*mhf2*^S59A^-*kanMX6 ura4-D18 his3-D1 leu1-32 arg3-D4* | This study |
| MCW6379 | *h^+^* | *mhf2*∆::*mhf2*^Q83A^-*kanMX6 ura4-D18 his3-D1 leu1-32 arg3-D4* | This study |
| MCW6367 | *h^-^* | *mhf2*∆::*mhf2*^D87A^-*kanMX6 ura4-D18 his3-D1 leu1-32 arg3-D4* | This study |
| FO808 | *h^-^* | *ura4-D18 his3-D1 leu1-32 arg3-D4* | Lab strain |
| MCW4708 | *h^-^* | *fml1*∆::*hphMX4 ura4-D18 his3-D1 leu1-32 arg3-D4* | This study |
| MCW6319 | *h^-^* | *mhf2*∆::*mhf2*^D87A^-*kanMX6 ura4-D18 his3-D1 leu1-32 arg3-D4*  *ade6-M210/M216* | This study |
| MCW6570 | *h^-^* | *fml1*∆::*hphMX4 mhf2*∆::*mhf2*^D87A^-*kanMX6 ura4-D18 his3-D1 leu1-32 arg3-D4 ade6-M210/M216* | This study |
| MCW5846 | *h^+^* | *mhf1*∆::*mhf1*-*GFP-kanMX6 mis6*∆::*mis6^+^*-*mCherry-ura4^+^ ura4-D18 leu1-32 his3-D1 arg3-D4* | (Bhattacharjee et al., 2013) |
| MCW6845 | *h^+^* | *mhf2*∆:: *natMX4 mhf1*∆::*mhf1*-*GFP-kanMX6 mis6*∆::*mis6^+^*-*mCherry-ura4^+^ ura4-D18 leu1-32 his3-D1 arg3-D4* | This study |
| MCW6884 | *h^-^* | *mhf2*∆::*mhf2*^D87A^-*hphMX4 mhf1*∆::*mhf1*-*GFP-kanMX6 mis6*∆::*mis6^+^*-*mCherry-ura4^+^ ura4-D18 leu1-32 his3-D1 arg3-D4* | This study |
| MCW5963 | *h^+^* | *fml1*∆::*hphMX4 mhf1*∆::*mhf1*-*GFP-kanMX6 mis6*∆::*mis6^+^*-*mCherry-ura4^+^ ura4-D18 leu1-32 his3-D1 arg3-D4* | (Bhattacharjee et al., 2013) |
| MCW6321 | *h^+^* | *mhf2*∆::*mhf2*^D87A^-*kanMX6 ura4-D18 his3-D1 leu1-32 arg3-D4* | This study |
| MCW7057 | *h^+^* | *fml1*∆::*fml1*^AAA^-*natMX4 mhf2*∆::*mhf2*^D87A^-*kanMX6 ura4-D18 his3-D1 leu1-32 arg3-D4* | This study |
| MCW9616 | *h^-^* | *fml1*∆::*fml1*^+^-*13Myc*-*natMX4 mhf1*∆::*mhf1*-*GFP-kanMX6 ura4-D18 leu1-32 his3-D1 arg3-D4* | This study |
| MCW9593 | *h^-^* | *fml1*∆::*fml1*^+^-*13Myc*-*natMX4 mhf1*∆::*mhf1*-*GFP-kanMX6 mhf2*∆::*mhf2*^D87A^-*hphMX4 ura4-D18 leu1-32 his3-D1 arg3-D4* | This study |
| MCW9594 | *h^-^* | *fml1*∆::*fml1*^AAA^-*13Myc*-*natMX4 mhf1*∆::*mhf1*-*GFP-kanMX6 ura4-D18 leu1-32 his3-D1 arg3-D4* | This study |
| MCW9595 | *h^-^* | *fml1*∆::*fml1*^AAA^-*13Myc*-*natMX4 mhf1*∆::*mhf1*-*GFP-kanMX6 mhf2*∆::*mhf2*^D87A^-*hphMX4 ura4-D18 leu1-32 his3-D1 arg3-D4* | This study |
| MCW9220 | *h^+^* | *mhf2*∆::*mhf2*^D87A^-*kanMX6 ade6-M375 int*::*pUC8/2000 bp spacer/his3^+^/RTS1-AO/ade6-L469 ura4-D18 his3-D1 leu1-32 arg3-D4* | This study |
| MCW9283 | *h^+^* | *fml1*∆::*fml1*^AAA^-*natMX4 ade6-M375 int*::*pUC8/2000 bp spacer/his3^+^/RTS1-AO/ade6-L469 ura4-D18 his3-D1 leu1-32 arg3-D4* | This study |
| MCW9269 | *h^+^* | *mhf2*∆::*mhf2*^D87A^-*kanMX6 fml1*∆::*fml1*^AAA^-*natMX4 ade6-M375 int*::*pUC8/2000 bp spacer/his3^+^/RTS1-AO/ade6-L469 ura4-D18 his3-D1 leu1-32 arg3-D4* | This study |
| MCW4406 | *h^+^* | *fml1*∆::*fml1*^+^-*13Myc*-*natMX4 ura4-D18 leu1-32 his3-D1 arg3-D4* | (Bhattacharjee et al., 2013) |
| MCW6977 | *h^-^* | *fml1*∆::*fml1*^∆C^-*13Myc*-*natMX4 ura4-D18 leu1-32 his3-D1 arg3-D4* | This study |
| MCW6980 | *h^+^* | *fml1*∆::*fml1*^AAA^-*13Myc*-*natMX4 ura4-D18 leu1-32 his3-D1 arg3-D4* | This study |
